# Intraocular Pressure Reduction Following Phacoemulsification in Patients with Exfoliation: A Systematic Review and Meta-Analysis

**DOI:** 10.3390/jcm13226774

**Published:** 2024-11-11

**Authors:** Konstantinos Benekos, Andreas Katsanos, Panagiotis Laspas, Iordanis Vagiakis, Anna-Bettina Haidich, Anastasios G. Konstas

**Affiliations:** 1 Ophthalmology Department, University of Ioannina, 45500 Ioannina, Greece; benekoskonstantinos@gmail.com (K.B.); katsanos@uoi.gr (A.K.); drlaspas@gmail.com (P.L.); 2 1st University Department of Ophthalmology, Aristotle University of Thessaloniki, 54124 Thessaloniki, Greece; jvag_@outlook.com; 3 Department of Hygiene, Social-Preventive Medicine and Medical Statistics, School of Medicine, Aristotle University of Thessaloniki, 54124 Thessaloniki, Greece; haidich@auth.gr

**Keywords:** exfoliation syndrome, exfoliative glaucoma, glaucoma, phacoemulsification, cataract surgery, intraocular pressure, IOP, ocular hypertension

## Abstract

**Objectives**: The objective of this systematic review and meta-analysis is to evaluate the existing evidence and estimate the impact of phacoemulsification and intraocular lens implantation on the intraocular pressure (IOP) of subjects with exfoliation syndrome (XFS) or exfoliative glaucoma (XFG). **Methods**: In July 2024, an in-depth literature review across three databases was undertaken. This study focused only on adult patients with exfoliation who had not undergone previous ocular surgery. The primary outcome of interest was the mean IOP reduction at 6 and 12 months after uncomplicated phacoemulsification surgery. **Results**: This meta-analysis included one randomized controlled trial and eight observational studies, comprising 220 patients at 6 months and 430 patients at the 12-month time point, respectively. The mean IOP reduction after surgery was 3.43 mmHg (95% CI: −4.77 to −2.09) after 6 months and 2.75 mmHg (95% CI: −4.24 to −1.26) after 12 months. In both time points, there was no heterogeneity (I^2^ = 0), but the certainty of evidence following the GRADE evaluation was very low. **Conclusions**: The present meta-analysis demonstrates that phacoemulsification can significantly reduce IOP in exfoliation subjects 6 and 12 months after surgery. Nevertheless, significant limitations in included studies do not allow a precise and certain estimate of the magnitude of postoperative IOP lowering in exfoliation patients. Additional research is needed to confirm these results.

## 1. Introduction

Exfoliation syndrome (XFS) is an age-related systemic condition in which abnormal extracellular fibrillar material progressively accumulates upon and within ocular tissues [1], leading to various ocular pathologies (e.g., cataract, glaucoma). XFS has been associated with a higher incidence of nuclear cataract, increased resistance to aqueous outflow, elevated intraocular pressure (IOP), and, consequently, a common form of secondary open-angle glaucoma (exfoliative glaucoma, XFG) [2]. Indeed, XFS is considered the most common identifiable cause of open-angle glaucoma [3]. The prognosis in XFG is typically worse than that of primary open-angle glaucoma (POAG), as it usually presents with worse IOP characteristics, greater optic nerve damage at diagnosis, and demonstrates a less favorable response to medical treatment [4,5]. Generally, eyes with XFS/XFG undergo phacoemulsification cataract surgery earlier and more often than eyes without exfoliation [6].

Today, IOP is the only known modifiable risk factor for glaucoma, and its control can delay or arrest glaucoma progression [7]. There is evidence suggesting that uncomplicated phacoemulsification can reduce IOP in eyes with POAG, [8] XFS, or XFG [9,10,11,12,13]. However, there is conflicting evidence concerning the magnitude and duration of this IOP-lowering effect in eyes with exfoliation. Some studies have reported that cataract extraction meaningfully decreases IOP up to 1 year after surgery [14], while others showed a much smaller reduction [10,12]. Considering the frequency with which cataract surgery is performed in patients with XFS/XFG, it is important to clarify the precise effect of this procedure on IOP. Thus, the aim of the present systematic review and meta-analysis was to evaluate existing data and estimate the effect of phacoemulsification on the IOP of eyes with exfoliation.

## 2. Materials and Methods

This study was conducted following the Preferred Reporting Items for Systematic Reviews and Meta-Analyses (PRISMA) guidelines [15] and registered at the PROSPERO registry under reference number CRD42024569109, which can be accessed at the following link: https://www.crd.york.ac.uk/prospero/display_record.php?RecordID=569109 (accessed on 10 August 2024).

Only studies that fulfilled the inclusion and exclusion criteria presented in detail in Table 1 were included. They should have enrolled adult patients with XFS or XFG, who had undergone uncomplicated phacoemulsification, and reported the IOP before, as well as 6 and 12 months after the procedure.

A thorough literature review was undertaken across the following three databases: Ovid Embase, PubMed/MEDLINE, and Cochrane Central Register of Controlled Trials (CENTRAL). Studies published until June 2024 were included. The literature search was performed without using any filters regarding the study type, and the search syntax for each database can be found in Appendix A. To ensure that all relevant studies were included, the reference lists of the identified narrative reviews, systematic reviews, and original studies were examined, too. After gathering all study reports, Deduplicator, a tool from the systematic review accelerator (SRA) suite [16] provided by Bond University, Australia, was used to remove the duplicates.

Two of the reviewers (KB and PL) independently examined the titles and abstracts of the papers retrieved from the database searches according to the preset inclusion and exclusion criteria. This screening process was conducted using Screenatron, a component of the SRA suite [16]. Disputatron, another tool in the SRA suite [16], was utilized to identify any differences in the screening process between the two reviewers. Discrepancies during this step were resolved by consensus.

The same reviewers (KB and PL) manually extracted data from the tables and full texts of the eligible studies. Details about the data extraction form can be found in Appendix B. Any disagreements between the two reviewers on the extracted data were resolved by consensus.

Along with data extraction, the reviewers (KB and PL) evaluated the risk of bias in the included studies. As only one arm of each study was analyzed, they were regarded as “before and after” studies, regardless of whether they were randomized controlled trials (RCTs) or observational studies. As a result, the risk of bias was assessed using the adapted “risk of bias in non-randomized studies of interventions” (ROBINS-I) tool [17].

The effect of cataract surgery on IOP was quantified using the mean difference (MD) and standard deviation (SD). The mean reduction in IOP at 6 and 12 months after phacoemulsification and the standard deviation (SD) values were extracted from the full texts of the included studies. If this data was not directly available, the preoperative and both 6- and 12-month postoperative mean IOP values and their SDs were extracted and used to calculate the mean IOP reduction and its SD at these time points. The calculations were performed using the equations outlined in the Cochrane Handbook [18]. In case of missing data, we contacted the study authors to collect the information needed for our systematic review and meta-analysis. If they did not respond within 2 weeks, the analysis was carried out using only the information provided in the study papers.

The meta-analysis was performed using a random effects model to estimate the overall effect. The analysis was carried out with the “metafor” package in R version 4.4. The level of statistical heterogeneity was evaluated using the I^2^ statistic and its confidence intervals. An I^2^ value exceeding 50% indicates a high degree of heterogeneity [18].

Regarding funnel plot assessment, reporting bias, heterogeneity, and poor methodological quality can cause asymmetry in these plots [19]. To investigate this asymmetry, we opted to use the test introduced by Egger et al. [20]. However, the decision was made not to use the funnel plot assessment with Egger’s test should the included studies be fewer than 10 [19].

Finally, the certainty of the body of evidence was assessed using the GRADE (Grading of Recommendations, Assessment, Development, and Evaluation) approach [21].

## 3. Results

### 3.1. Search Results

**Figure 1:**
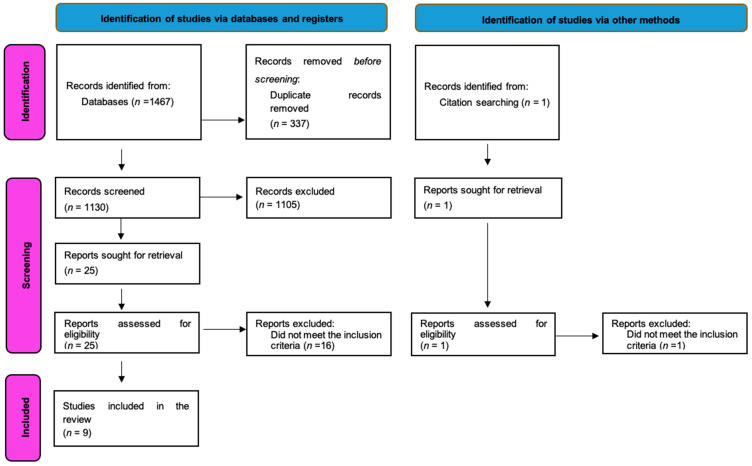
PRISMA flowchart.

The literature search was conducted in July 2024, and after duplicate removal, 1130 articles from three peer-reviewed databases and one study from the reference list screening of the relevant studies were retrieved (Figure 1).

The studies that were excluded after their full-text assessment and the reason for their exclusion are mentioned in Appendix C.

The present meta-analysis included nine studies, of which one was a randomized controlled trial (RCT) and eight were observational studies (Table 2). Two hundred twenty patients with XFS/XFG were included at the 6-month follow-up timepoint and 430 patients at the 12-month timepoint. The studies were selected according to the predefined inclusion and exclusion criteria described in Section 2. Each study had at least one group of patients with XFS, or XFG, who had undergone uncomplicated phacoemulsification, and only these groups were included in the analysis. From the study by Singleton et al. [12], we included and analyzed only the XFS group, as some patients from the XFG group had already undergone laser trabeculoplasty or another kind of glaucoma filtration surgery. The primary outcome of interest was to evaluate the average IOP reduction at 6- and 12 months following phacoemulsification in these patients.

### 3.2. Risk of Bias in the Included Studies

**Figure 2:**
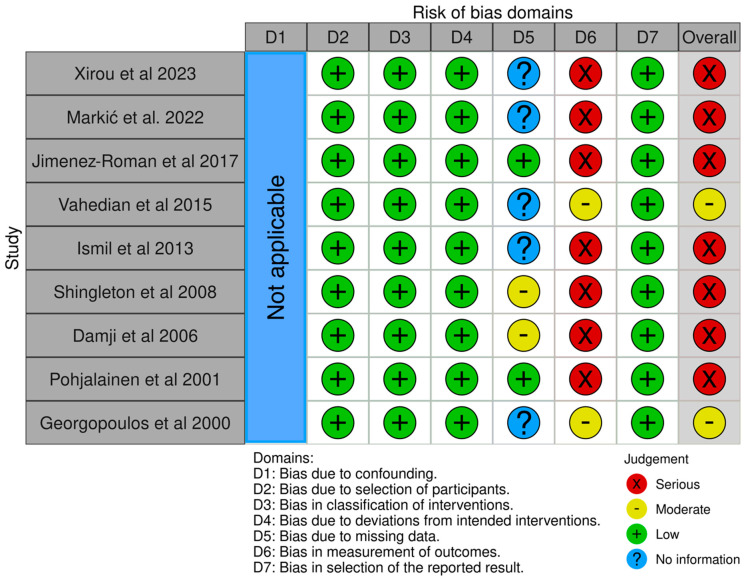
Risk of Bias [10,11,12,22,23,24,25,26,27].

The risk of bias in the studies included in this meta-analysis is presented in the Figure 2.

#### 3.2.1. Bias Due to Confounding

The “Bias due to confounding” domain in the ROBINS-I tool [17] is not applicable to this review since all participants in each study were assigned to the same group. All individuals received the same intervention (i.e., phacoemulsification); thus, baseline and time-varying confounding do not apply to this study.

#### 3.2.2. Bias Due to the Selection of Participants

The selection of participants in each study was not influenced by characteristics noted after the intervention began, and the follow-up period started simultaneously with the intervention.

#### 3.2.3. Bias in the Classification of Interventions

Phacoemulsification, a standardized surgical procedure, was well-defined in all the studies; thus, all studies were graded as having a low risk of bias in this domain.

#### 3.2.4. Bias Due to Deviations from the Intended Interventions

In the included studies, there were no deviations from the intended interventions beyond what would be expected in usual practice. As a result, they were assessed as having a low risk of bias in this domain.

#### 3.2.5. Bias Due to Missing Data

Most studies did not provide adequate information on missing data, as they did not mention whether the number of patients assessed at the last follow-up differed from that at baseline. Only four studies [10,12,24,27] reported patient loss during follow-up. The attrition rate was below 20% in two of them [24,27], and the missing data in these was unlikely to be related to the intervention or cause any issue with the outcome of interest of this meta-analysis. Thus, they were marked as having a low risk of bias in this domain. On the other hand, in the other two studies [10,12], much higher attrition was observed. The authors of these latter studies did not clarify why some patients did not attend the 6- or 12-month follow-up visits. Therefore, we assessed these two studies as having a moderate risk of bias in this domain.

#### 3.2.6. Bias in the Measurement of Outcomes

It is well-established that IOP measurements taken before and after phacoemulsification can be affected by topical medications [28]. None of the studies reviewed included a washout period to measure IOP without the influence of these drops, and this might have affected tonometry measurements. However, a few studies reported that the patients were kept on their preoperative IOP-lowering medications to reduce any treatment-related pressure changes [10,22,23]. Three studies included patients who did not require treatment as they did not have XFG [12,25,27]. Another source of bias in these studies, with the exception of the RCT by Georgopoulos et al. [11], was that the outcome assessors were not masked to the intervention. Moreover, only three studies reported that the IOP measurements were performed at least twice daily [11,24,25]. The absence of multiple measurements at baseline and follow-up visits could lead to bias due to the fluctuation of the IOP in XFS/XFG patients. Taking all into account, only two studies [11,25] were rated as having a moderate risk of bias, while the remaining studies were evaluated as having a high risk of bias (Table 3).

#### 3.2.7. Bias in the Selection of the Reported Result

Those studies did not have any preregistered protocol. However, there is no evidence that the reported analysis was chosen from among multiple possible analyses or based on the results. Thus, all studies were graded as having a low risk of bias in this domain.

### 3.3. Meta-Analysis

We performed the meta-analysis using a random-effects model to evaluate the impact of phacoemulsification on the IOP of patients with XFS or XFG. The available data was analyzed at two separate time points following the procedure: at 6 and 12 months.

#### 3.3.1. The Effect of Phacoemulsification on IOP at 6 Months

**Figure 3:**
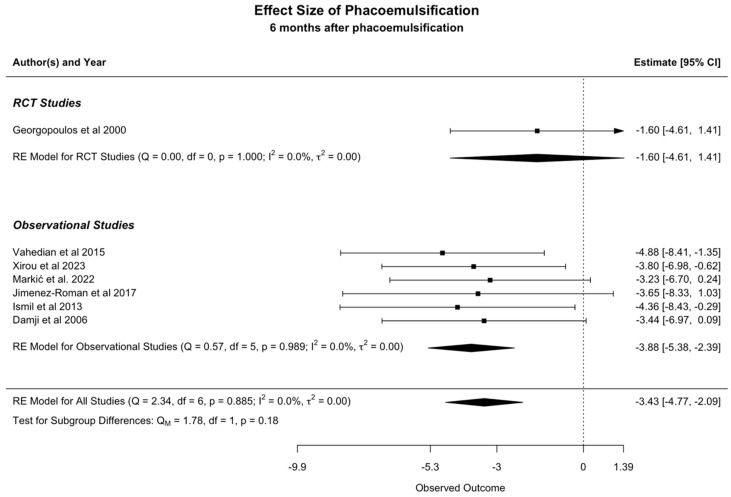
Meta-Analysis at 6 months [10,11,22,23,24,25,26].

The analysis of the available data showed a mean IOP reduction of 3.43 mmHg (95% CI: −4.77 to −2.09) six months after phacoemulsification (Figure 3). The observed I^2^ is equal to zero, indicating that statistical heterogeneity is low.

#### 3.3.2. The Effect of Phacoemulsification on IOP at 12 Months

**Figure 4:**
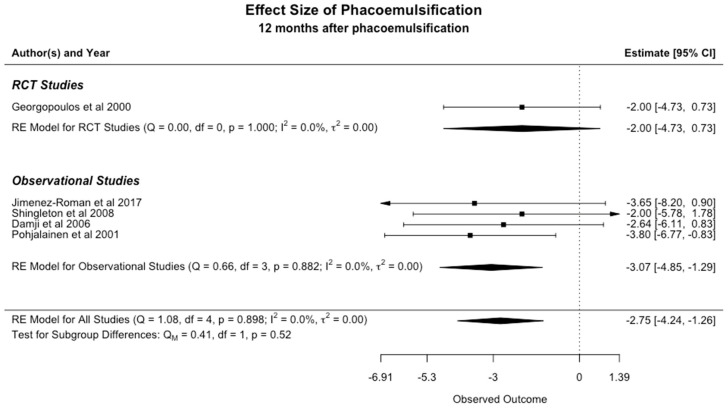
Meta-Analysis at 12 months [10,11,12,24,27].

The analysis of the included studies revealed that the average IOP decreased by 2.75 mmHg (95% CI: −4.24 to −1.26) 12 months following phacoemulsification (Figure 4). The observed I^2^ value is zero, which indicates that there is no significant statistical heterogeneity.

### 3.4. Certainty of Evidence in Each Subgroup

**Figure 5:**
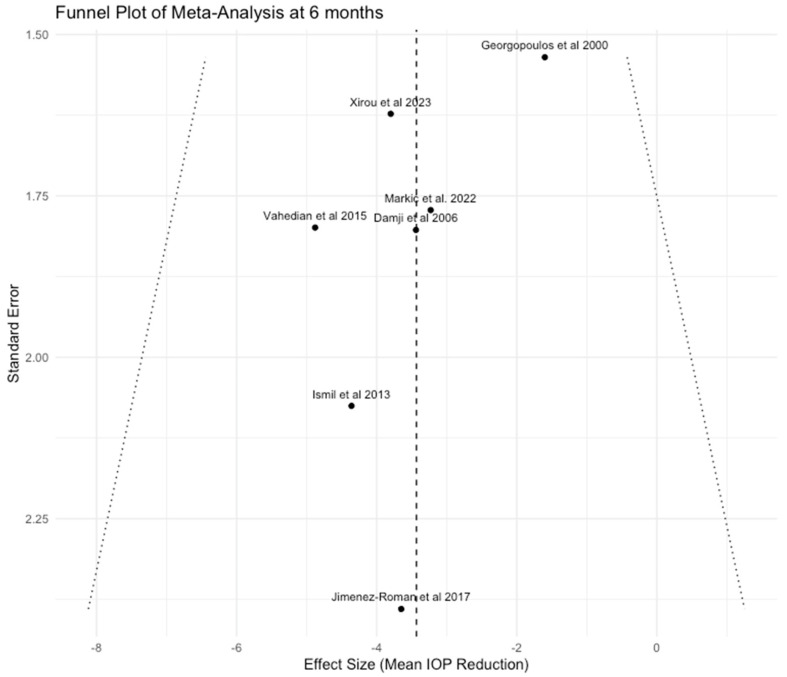
Funnel plot of Meta-Analysis at 6 months [10,11,14,22,23,25,26].

**Figure 6:**
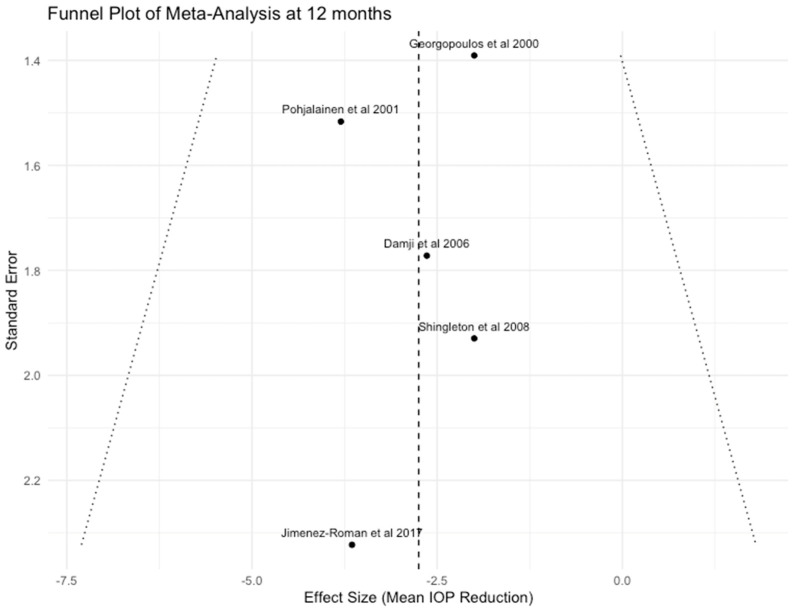
Funnel plot of Meta-Analysis at 12 months [10,11,12,24,27].

For both analyses at 6 and 12 months, we downgraded the certainty of the evidence by two levels because all the included studies, except for two [11,25], were graded as having a high risk of bias using the ROBINS-I tool [17]. In addition, the relatively small sample size and wide confidence intervals of the included studies may also lower the accuracy of the metanalysis results, leading us further to downgrade the level of evidence by one level. Lastly, the visual inspection of the funnel plots (Figure 5 and Figure 6) indicated that some small studies had reported a significant IOP reduction higher than 3 mmHg after phacoemulsification, but on the other hand, studies with lower or no effects on IOP are totally absent or may not have been published yet. This raises suspicions about the presence of publication bias, and thus, we chose to decrease the quality of evidence to yet another level. Considering all the above and having in mind that most studies included in our analysis were observational in design, which by default sets the initial quality of evidence to “Low”, we assessed the overall certainty of the evidence as “Very Low” at both time points (Table 4).

## 4. Discussion

The current systematic review and meta-analysis focused on the impact of phacoemulsification as a standalone procedure on the IOP at 6 and 12 months after surgery in patients with XFG or XFS. Our search strategy across the mentioned databases was, by design, extensive with almost no restrictions to avoid the chances of missing relevant studies. To assess the overall impact of phacoemulsification on IOP in these patients, we only analyzed the phacoemulsification groups of the eligible studies.

Our meta-analysis concluded that phacoemulsification can significantly reduce IOP in eyes with exfoliation. The analysis of available published data indicated that IOP decreased by 3.43 mmHg (95% CI: −4.77 to −2.09) at 6 months and 2.75 mmHg (95% CI: −4.24 to −1.26) at 12 months following the procedure. Of note, the low heterogeneity observed at both time points suggests a high degree of consistency across studies, strengthening the reliability of our findings. Visual inspection of the funnel plots showed that mainly small studies reporting a reduction in IOP greater than 3 mmHg following phacoemulsification were present, which could imply that those showing lower or minimal effects might remain unpublished. However, given the small number of studies and the limitations of funnel plot assessment [29], we cannot draw a definitive conclusion about the presence or absence of publication or reporting bias. Egger’s test was not performed following the recommendations that it should not be used if there are fewer than 10 studies in the analysis [19].

It is noteworthy that the findings of this meta-analysis, which was focused on exfoliation subjects, are consistent with another recent meta-analysis [8] that assessed the impact of phacoemulsification on IOP, mainly in POAG patients. Six months after surgery, the results in XFS/XFG subjects are similar to those seen in POAG patients at 12 months. However, at 12 months, the effect of phacoemulsification in exfoliation patients seems to be lower than that seen in patients with POAG. We hypothesize that this difference might simply be due to chance or could be explained by the possibility that exfoliation material, which was washed away during phacoemulsification, may slowly re-accumulate in the trabecular meshwork.

Previous meta-analyses have also explored how cataract extraction can affect IOP in patients with XFS/XFG [9,13]. The most recent ones have some limitations regarding their analysis and the studies they included. For example, the meta-analysis by Masis et al. [9] analyzed data from the included studies at eye level, which raises concerns about whether studies that enrolled both eyes from some patients were included. Indeed, one [30] of the four studies included in the meta-analysis by Masis et al. [9] enrolled two eyes of some patients in the phacoemulsification group. The same issue can be noticed in the recent meta-analysis by Pasquali et al. [13], which has also included a study [31] that enrolled both eyes of several patients. It is generally accepted that observations from one eye of a patient may be similar to those from the other eye of the same individual [32]. However, this assumption does not apply when comparing eyes from different patients. Ignoring this eye-to-eye correlation can lead to misleading results [33]. Another limitation of that paper [13] was the incomplete evaluation of the risk of bias and quality of each study, which is a crucial component of a systematic review and meta-analysis.

Although the results of the two abovementioned meta-analyses are in accordance with our findings, it is worth mentioning that the mean IOP reduction reported in these studies is approximately 2 mmHg higher than that observed in the current analysis. This difference might be due to the inclusion of a study by Jacobi et al. [14] in their analysis but not in ours. Among all studies that examined IOP lowering in eyes with exfoliation, Jacobi et al. [14] reported the highest mean IOP reduction at both 6 and 12 months after phacoemulsification. This may occur because the IOP-reducing effect of interventions such as phacoemulsification can be influenced by the phenomenon of regression to the mean, especially when preoperative values are high [34], just as in the study by Jacobi et al. [14]. Generally, higher preoperative values are strong predictors of higher overall IOP reduction, as has been shown in several studies [34,35,36]. We excluded the study by Jacobi et al. [14] because they had included one patient with complications and vitreous loss in the phacoemulsification arm, and thus, it did not fulfill the inclusion criteria of our analysis.

A key limitation of our meta-analysis is that all included studies did not employ a washout period before IOP measurements. Li et al. [28] suggested that each IOP-lowering medication might have a different ocular hypotensive effect, ranging from around 2 to almost 6 mmHg. Therefore, changes in the number or type of topical medications between baseline and post-phacoemulsification follow-up could significantly impact the actual calculated IOP-lowering effect of phacoemulsification. To mitigate this phenomenon, three studies in our meta-analysis maintained the same antiglaucoma medications after surgery [10,22,23]. However, the remaining studies, which included XFG patients already on IOP-lowering medications, reported a reduction in the number of eyedrops used [11,24,26]. Despite the fact that this reduction in eyedrop use may limit our ability to accurately calculate and quantify the exact ocular hypotensive effect of phacoemulsification, the decreased need for IOP-lowering medications postoperatively strongly suggests that the IOP is, indeed, reduced after phacoemulsification. The concerns about pre- and postoperative medications were not relevant for three of the studies analyzed, as they had only included patients with XFS syndrome who did not require IOP-lowering medications [12,25,27].

On the other hand, a key strength of the current meta-analysis is the fact that it is controlled for inter-eye correlation by analyzing only one eye per patient. We also excluded studies with complicated cases so that our results portray the impact of routine, uncomplicated phacoemulsification surgery on IOP. Thus, the results are applicable to typical clinical settings. Despite these exclusion criteria, our analysis involved the largest number of studies compared to other meta-analyses on cataract extraction in eyes with exfoliation. Finally, we utilized the ROBINS-I tool [17] to assess the risk of bias in the included studies, following the instructions outlined in the Cochrane Handbook [18].

## 5. Conclusions

In eyes with exfoliation, IOP is reduced after uncomplicated phacoemulsification surgery by 3.43 mmHg (95% CI: −4.77 to −2.09) at 6 months and 2.75 mmHg (95% CI: −4.24 to −1.26) at 12 months post-surgery. Nonetheless, the GRADE approach [21] ranked the certainty of the evidence of these results as “Very Low”, suggesting that there is little confidence in them. Our analysis addressed the inter-eye correlation and excluded complicated cases, but the potential effects of pre- and postoperative medication changes on IOP reduction remain a concern. More controlled studies with appropriate washout periods, diurnal IOP measurements at baseline and follow-up visits, and consistent monitoring of medication use are needed to provide more reliable evidence and validate our results in the future.

## Figures and Tables

**Table 1:** Inclusion and exclusion criteria.

	Inclusion Criteria	Exclusion Criteria
Study characteristics	Phacoemulsification arms Language: English, French, German	Literature reviews Case reports Conference abstracts
Participants	Adult patients with XFS or XFG Only one eye per patient was included	History of trabeculectomy or other ocular surgery for other ocular diseases Sample size less <10 in the phacoemulsification arm
Intervention	Uncomplicated phacoemulsification	Any other lens extraction surgery
Outcomes	Mean difference in IOP at 6 and 12 months after phacoemulsification compared to baseline	

**Table 2:** Baseline characteristics of the included studies.

Study	Type of Study	Participants in XFS/XFG+ Phaco Arm at Baseline	Men/Women in XFS/XFG+ Phaco Arm at Baseline	Mean Age in XFS/XFG+ Phaco Arm at Baseline (SD)	Participants Analyzed XFS/XFG+ Phaco Arm (One Eye per Participant)	Max Follow-Up Period	Diagnosis of Patients Analyzed in XFS/XFG+ Phaco Arm	Mean Preoperative IOP of Patients on XFS/XFG + Phaco Arm (SD)
Xirou et al., 2023 [22]	Observational	25	12/13	66.1 (4.2)	10 (at 6 months)	6 m	XFG	18.1 (1.91)
Markić et al., 2022 [23]	Observational	31	20/11	76 (6)	31 (at 6 months)	6 m	XFG	16.27 (3.08)
Jimenez-Roman et al., 2017 [24]	Observational	44	17/27	75.86 (7.79)	39 (at 6 months)	12 m	XFG	17.9 (5.8 *)
					35 (at 12 months)			
Vahedian et al., 2015 [25]	Observational	68	42/26	68.84 (6.62)	68 (at 6 months)	6 m	XFS	17.45 (3.32)
Ιsmil et al., 2013 [26]	Observational	11	NI	62.24 (14.53)	11 (at 6 months)	6 m	XFG	15.27 (4.38)
Shingleton et al., 2008 [12]	Observational	882	241/641	77.6	311 (at 12 months)	10 y	XFS	16.4 (3.6)
Damji et al., 2006 [10]	Observational	71	20/51	75.98 (6.74)	48 (at 6 months)	24 m	XFS and XFG	17.6 (3.24)
					52 (at 12 months)			
Pohjalainen et al., 2001 [27]	Observational	24	NI	76.3 (11.2)	19 (at 12 months)	2.7 y	XFS	16.3 (2.7)
Georgopoulos et al., 2000 [11]	RCT	14	NI	65.8 (4.4)	13 (at 6 and 12 months)	18 m	XFG	18.7 (1.84)

XFS = exfoliation syndrome, XFG = exfoliative glaucoma, IOP = intraocular pressure, NI = no information, phaco = phacoemulsification, SD = standard deviation, RCT = randomized controlled trial, m = months, y = years, *: extracted from plot.

**Table 3:** Bias in the measurement of outcomes.

Studies	Patients Kept on Their Preoperative IOP-Lowering Medications	Masked Assessors	IOP Measurements at Least Twice Daily	Overall Bias in Measurement of Outcomes
Xirou et al., 2023 [22]	Yes	No	No	Serious
Markić et al., 2022 [23]	Yes	No	No	Serious
Jimenez-Roman et al., 2017 [24]	No	No	Yes	Serious
Vahedian et al., 2015 [25]	No medications	No	Yes	Moderate
Ιsmil et al., 2013 [26]	No	No	No	Serious
Shingleton et al., [12]	No medications	No	No	Serious
Damji et al., 2006 [10]	Yes	No	No	Serious
Pohjalainen et al., 2001 [27]	No medications	No	No	Serious
Georgopoulos et al., 2000 [11]	No	Yes	Yes	Moderate

**Table 4:** Summary of findings and certainty assessment.

Number of Participants Analyzed in XFS/XFG+ Phaco Arms (Studies)	Study Design	Risk of Bias	Inconsistency	Indirectness	Imprecision	Publication Bias	Mean Change in IOP from Baseline (95% CI)	Certainty of the Evidence (GRADE)
**At 6 months after phacoemulsification**								
220 (7)	Mainly observational	Very Serious	Not serious	Not serious	Serious	Serious	−3.43 mmHg (−4.77 to −2.09)	Very low ⊕◯◯◯
**At 12 months after phacoemulsification**								
430 (5)	Observational	Very Serious	Not serious	Not serious	Serious	Serious	−2.75 mmHg (−4.24 to −1.26)	Very low ⊕◯◯◯

CI = confidence interval, GRADE = Grading of Recommendations, Assessment, Development, and Evaluation, XFS = exfoliation syndrome, XFG = exfoliative glaucoma, IOP = intraocular pressure.

## Appendix A

### Appendix A.1. PubMed/MEDLINE

#### Search Syntax

#1 exfoliation syndrome [All Fields]
#2 exfoliation syndrome [MeSH Terms]
#3 XFS [All Fields]
#4 PEX [All Fields]
#5 Pseudoexfoliation syndrome [All Fields]
#6 Pseudoexfoliation syndrome [MeSH Terms]
#7 XFG [All Fields]
#8 PXF [All Fields]
#9 exfoliative Glaucoma [All Fields]
#10 exfoliative Glaucoma [MeSH Terms]
#11 Pseudoexfoliative Glaucoma [All Fields]
#12 Pseudoexfoliative Glaucoma [MeSH Terms]
#13 Capsular glaucoma [All Fields]
#14 1 or 2 or 3 or 4 or 5 or 6 or 7 or 8 or 9 or 10 or 11 or 12 or 13
#15 cataract
#16 cataract [MeSH Terms]
#17 cataracts [All Fields]
#18 cataracts [MeSH Terms]
#19 cataractic [All Fields]
#20 cataractous [All Fields]
#21 15 or 16 or 17 or 18 or 19 or 20
#22 phacoemulsification [All Fields]
#23 phacoemulsification [MeSH Terms]
#24 phacoemulsifications [All Fields]
#25 phacoemulsifications [MeSH Terms]
#26 cataract extraction [All Fields]
#27 cataract extraction [MeSH Terms]
#28 cataract surgery [All Fields]
#29 cataract surgery [MeSH Terms]
#30 #phakoemulsif* [All Fields]
#31 phako-emulsif* [All Fields]
#32 phacoemulsif* [All Fields]
#33 phaco-emulsif* [All Fields]
#34 lens extraction [All Fields]
#35 phaco [All Fields]
#36 OR #19-32
#37 intraocular pressure [All Fields]
#38 intraocular pressure [MeSH Terms]
#39 IOP [All Fields]
#40 34 OR 35 OR 36
#41 14 AND 21 AND 33 AND 37

### Appendix A.2. Ovid Embase

#### Search Syntax

#1 exfoliation syndrome.mp. or pseudoexfoliation/
#2 pseudoexfoliation/ or Pseudoexfoliation syndrome.mp.
#3 XFS.mp.
#4 PEX.mp.
#5 Pseudoexfoliative Glaucoma.mp. or pseudoexfoliation/
#6 exfoliative Glaucoma.mp. or pseudoexfoliation/
#7 XFG.mp.
#8 PXF.mp.
#9 PES.mp.
#10 Capsular glaucoma.mp. or pseudoexfoliation/
#11 1 or 2 or 3 or 4 or 5 or 6 or 7 or 8 or 9 or 10
#12 phacoemulsification needle/ or phacoemulsification/ or phacoemulsification.mp.
#13 cataract extraction.mp. or cataract extraction/
#14 cataract surgery.mp. or cataract extraction/
#15 phakoemulsif*.mp. or cataract extraction/
#16 cataract extraction/ or phacoemulsification/ or phacoemulsif*.mp.
#17 cataract extraction/ or phacoemulsification/ or phaco.mp. or cataract/
#18 12 or 13 or 14 or 15 or 16 or 17
#19 subcapsular cataract/ or cataract/ or cataract.mp. or senile cataract/ or traumatic cataract/
#20 cataract/ or cataractous.mp.
#21 cataract/ or cataractic.mp.
#22 19 or 20 or 21
#23 intraocular pressure.mp. or intraocular pressure/
#24 IOP.mp.
#25 23 or 24
#26 11 and 18 and 22 and 25

### Appendix A.3. EBM Reviews—Cochrane Central Register of Controlled Trials

#### Search Syntax

#1 exfoliation syndrome.mp. or pseudoexfoliation/
#2 pseudoexfoliation/ or Pseudoexfoliation syndrome.mp.
#3 XFS.mp.
#4 PEX.mp.
#5 Pseudoexfoliative Glaucoma.mp. or pseudoexfoliation/
#6 exfoliative Glaucoma.mp. or pseudoexfoliation/
#7 XFG.mp.
#8 PXF.mp.
#9 PES.mp.
#10 Capsular glaucoma.mp. or pseudoexfoliation/
#11 1 or 2 or 3 or 4 or 5 or 6 or 7 or 8 or 9 or 10
#12 phacoemulsification needle/ or phacoemulsification/ or phacoemulsification.mp.
#13 cataract extraction.mp. or cataract extraction/
#14 cataract surgery.mp. or cataract extraction/
#15 phakoemulsif*.mp. or cataract extraction/
#16 cataract extraction/ or phacoemulsification/ or phacoemulsif*.mp.
#17 cataract extraction/ or phacoemulsification/ or phaco.mp. or cataract/
#18 12 or 13 or 14 or 15 or 16 or 17
#19 subcapsular cataract/ or cataract/ or cataract.mp. or senile cataract/ or traumatic cataract/
#20 cataract/ or cataractous.mp.
#21 cataract/ or cataractic.mp.
#22 19 or 20 or 21
#23 intraocular pressure.mp. or intraocular pressure/
#24 IOP.mp.
#25 23 or 24
#26 11 and 18 and 22 and 25

## Appendix B

Data extraction list

From each study, we extracted the information described below:

- First author and year
- Title of paper
- Study link or DOI
- Lead author contact details
- The country in which the study conducted
- Study design
- Follow-up period
- Study funding sources
- Possible conflicts of interest for study authors
- 1 eye per patient in the phaco arm (Yes/No)
- Total number of participants at baseline
- Total number of eyes at baseline
- Participants in XFS/XFG+ phaco group at baseline
- Number of eyes in XFS/XFG+ phaco group at baseline
- Participants analyzed XFS/XFG + phaco arm at 6 and 12 months
- Number of eyes in XFS/XFG+ phaco group at baseline at 6 and 12 months
- Diagnosis (XFS or XFG)
- Mean age in XFS/XFG+ phaco group at baseline (SD)
- Males/ Females in XFS/XFG+ phaco group at baseline
- Mean IOP pre-op in XFS/XFG+ phaco group (SD)
- Number of medications at baseline in XFS/XFG+ phaco group (SD)
- IOP at 6 months in XFS/XFG+ phaco group (SD)
- Mean IOP reduction at 6 months in XFS/XFG+ phaco group (SD)
- Number of medications at 6 months in XFS/XFG+ phaco group (SD)
- IOP at 12 months in XFS/XFG+ phaco group (SD)
- Mean IOP reduction at 12 months in XFS/XFG+ phaco group (SD)
- Number of medications at 12 months in XFS/XFG+ phaco group (SD)

## Appendix C

Characteristics of the excluded studies at full-text evaluation

**Study**	**Reason for Exclusion**
1. Abdelghany et al., J Ophthalmol, 2019 [37]	Νοt 1 eye per patient
2. Burgmüller et al., Graefe’s Archive for Clinical and Experimental Ophthalmology, 2018 [38]	No IOP in baseline and no IOP in 6 or 12 months
3. Cimetta et al., European Journal of Ophthalmology, 2008 [39]	Ranges are mentioned as measures of variation instead of CI. Ranges should not be used to estimate SDs according to the Cochrane Handbook.
4. Haripriya et al., Ophthalmology, 2018 [40]	Cases with complications are included in the tested groups
5. Jacobi et al., Archives of ophthalmology, 1999 [14]	1 patient with vitreous loss
6. Kang et al., Scientific reports, 2024 [41]	Baseline IOP is not referred to the IOP before phacoemulsification
7. Merkur et al., Journal of Cataract and Refractive Surgery, 2001 [31]	1 capsular bag dehiscence in the XFS group
8. Mierzejewski et al., KlinikaOczna, 2008 [30]	In 1 patient, rupture of the posterior capsule occurred, with vitreous prolapse.
9. Perasalo et al., Acta Ophthalmologica Scandinavica, 1997 [42]	Not only XFG but POAG patients, too. Not 1 eye per patient
10. Rodriguez-Una et al., Journal of Glaucoma, 2023 [43]	Not 1 eye per patient in the symmetric group.In the asymmetric group, some patients underwent glaucoma laser treatment or filtering surgery after phacoemulsification
11. Pose-Bazarra et al., Eye (London, England), 2022 [44]	Less than 10 patients in the phacoemulsification arm
12. Shingleton et al., Journal of Cataract and Refractive Surgery, 2008 [45]	Several eyes in XFS group had extracapsular cataract extraction.
13. Shingleton et al., Journal of cataract and refractive surgery, 2003 [46]	Νοt 1 eye per patient
14. Tekcan et al., International Ophthalmology, 2022 [47]	Νοt 1 eye per patient
15. Zarei et al., Eye (London, England), 2022 [48]	No purely XFS/ XFG patients in the phacoemulsification group
16. Yalvac et al., Ophthalmic Surg Lasers. 1997 [49]	Patients with diagnosis other than XFG were also included in the phaco group
17. Wirbelauer et al., Ophthalmic Surg Lasers, 1998 [50]	In 1 patient in the XFG group, rupture of the posterior capsule occurred, with vitreous loss.

## References

[1] Ritch R., Schlotzer-Schrehardt U. (2001). Exfoliation syndrome. Surv. Ophthalmol..

[2] Naumann G.O., Schlotzer-Schrehardt U., Kuchle M. (1998). Pseudoexfoliation syndrome for the comprehensive ophthalmologist. Intraocular Syst. Manif. Ophthalmol..

[3] Ritch R. (1994). Exfoliation syndrome-the most common identifiable cause of open-angle glaucoma. J. Glaucoma.

[4] Conway R.M., Schlotzer-Schrehardt U., Kuchle M., Naumann G.O. (2004). Pseudoexfoliation syndrome: Pathological manifestations of relevance to intraocular surgery. Clin. Exp. Ophthalmol..

[5] Hollo G., Katsanos A., Konstas A.G. (2015). Management of exfoliative glaucoma: Challenges and solutions. Clin. Ophthalmol..

[6] Drolsum L., Ringvold A., Nicolaissen B. (2007). Cataract and glaucoma surgery in pseudoexfoliation syndrome: A review. Acta Ophthalmol. Scand..

[7] Kass M.A., Heuer D.K., Higginbotham E.J., Johnson C.A., Keltner J.L., Miller J.P., Parrish R.K., Wilson M.R., Gordon M.O. (2002). The Ocular Hypertension Treatment Study: A randomized trial determines that topical ocular hypotensive medication delays or prevents the onset of primary open-angle glaucoma. Arch. Ophthalmol..

[8] Benekos K., Katsanos A., Haidich A.B., Dastiridou A., Nikolaidou A., Konstas A.G. (2024). The Effect of Phacoemulsification on the Intraocular Pressure of Patients With Open Angle Glaucoma: A Systematic Review and Meta-Analysis. J. Glaucoma.

[9] Masis M., Mineault P.J., Phan E., Lin S.C. (2018). The role of phacoemulsification in glaucoma therapy: A systematic review and meta-analysis. Surv. Ophthalmol..

[10] Damji K.F., Konstas A.G.P., Liebmann J.M., Hodge W.G., Ziakas N.G., Giannikakis S., Mintsioulis G., Merkur A., Pan Y., Ritch R. (2006). Intraocular pressure following phacoemulsification in patients with and without exfoliation syndrome: A 2 year prospective study. Br. J. Ophthalmol..

[11] Georgopoulos G.T., Chalkiadakis J., Livir-Rallatos G., Theodossiadis P.G., Theodossiadis G.P. (2000). Combined clear cornea phacoemulsification and trabecular aspiration in the treatment of pseudoexfoliative glaucoma associated with cataract. Graefe’s Arch. Clin. Exp. Ophthalmol..

[12] Shingleton B.J., Laul A., Nagao K., Wolff B., O’Donoghue M., Eagan E., Flattem N., Desai-Bartoli S. (2008). Effect of phacoemulsification on intraocular pressure in eyes with pseudoexfoliation: Single-surgeon series. J. Cataract. Refract. Surg..

[13] Pasquali A., Varano L., Ungaro N., Tagliavini V., Mora P., Goldoni M., Gandolfi S. (2024). Does Cataract Extraction Significantly Affect Intraocular Pressure of Glaucomatous/Hypertensive Eyes? Meta-Analysis of Literature. J. Clin. Med..

[14] Jacobi P.C., Dietlein T.S., Krieglstein G.K. (1999). Comparative study of trabecular aspiration vs trabeculectomy in glaucoma triple procedure to treat pseudoexfoliation glaucoma. Arch. Ophthalmol..

[15] Page M.J., McKenzie J.E., Bossuyt P.M., Boutron I., Hoffmann T.C., Mulrow C.D., Shamseer L., Tetzlaff J.M., Akl E.A., Brennan S.E. (2021). The PRISMA 2020 statement: An updated guideline for reporting systematic reviews. BMJ.

[16] Clark J., Glasziou P., Del Mar C., Bannach-Brown A., Stehlik P., Scott A.M. (2020). A full systematic review was completed in 2 weeks using automation tools: A case study. J. Clin. Epidemiol..

[17] Sterne J.A., Hernan M.A., Reeves B.C., Savovic J., Berkman N.D., Viswanathan M., Henry D., Altman D.G., Ansari M.T., Boutron I. (2016). ROBINS-I: A tool for assessing risk of bias in non-randomised studies of interventions. BMJ.

[18] Higgins J.P.T., Thomas J., Chandler J., Cumpston M., Li T., Page M.J., Welch V.A. (2024). Cochrane Handbook for Systematic Reviews of Interventions Version 6.5 (Updated August 2024).

[19] Sterne J.A., Sutton A.J., Ioannidis J.P., Terrin N., Jones D.R., Lau J., Carpenter J., Rucker G., Harbord R.M., Schmid C.H. (2011). Recommendations for examining and interpreting funnel plot asymmetry in meta-analyses of randomised controlled trials. BMJ.

[20] Egger M., Davey Smith G., Schneider M., Minder C. (1997). Bias in meta-analysis detected by a simple, graphical test. BMJ.

[21] Balshem H., Helfand M., Schunemann H.J., Oxman A.D., Kunz R., Brozek J., Vist G.E., Falck-Ytter Y., Meerpohl J., Norris S. (2011). GRADE guidelines: 3. Rating the quality of evidence. J. Clin. Epidemiol..

[22] Xirou V., Xirou T., Siganos C., Ntonti P., Georgakopoulos C., Stavrakas P., Makri O.E., Kanakis M., Tsapardoni F., Fragkoulis I. (2023). Impact of Cataract Surgery on IOP and Ocular Structures in Normotensive Patients and Primary and Exfoliation Open-Angle Glaucoma Patients. Clin. Ophthalmol..

[23] Markic B., Mavija M., Smoljanovic-Skocic S., Popovic M.T., Burgic S.S. (2022). Predictors of intraocular pressure change after cataract surgery in patients with pseudoexfoliation glaucoma and in nonglaucomatous patients. Vojnosanit. Pregl..

[24] Jimenez-Roman J., Lazcano-Gomez G., Martínez-Baez K., Turati M., Gulías-Cañizo R., Hernández-Zimbrón L.F., Ochoa-De la Paz L., Zamora R., Gonzalez-Salinas R. (2017). Effect of phacoemulsification on intraocular pressure in patients with primary open angle glaucoma and pseudoexfoliation glaucoma. Int. J. Ophthalmol..

[25] Vahedian Z., Salmanroghani R., Fakhraie G., Moghimi S., Eslami Y., Zarei R., Mohammadi M. (2015). Pseudoexfoliation syndrome: Effect of phacoemulsification on intraocular pressure and its diurnal variation. J. Curr. Ophthalmol..

[26] Ismi T., Yilmaz A. (2013). Effects of cataract surgery on intraocular pressure in patients with and without glaucoma. Turk. Oftalmoloiji Derg..

[27] Pohjalainen T., Vesti E., Uusitalo R.J., Laatikainen L. (2001). Intraocular pressure after phacoemulsification and intraocular lens implantation in nonglaucomatous eyes with and without exfoliation. J. Cataract. Refract. Surg..

[28] Li T., Lindsley K., Rouse B., Hong H., Shi Q., Friedman D.S., Wormald R., Dickersin K. (2016). Comparative Effectiveness of First-Line Medications for Primary Open-Angle Glaucoma: A Systematic Review and Network Meta-analysis. Ophthalmology.

[29] Lau J., Ioannidis J.P., Terrin N., Schmid C.H., Olkin I. (2006). The case of the misleading funnel plot. BMJ.

[30] Mierzejewski A., Eliks I., Kaluzny B., Zygulska M., Harasimowicz B., Kaluzny J.J. (2008). Cataract phacoemulsification and intraocular pressure in glaucoma patients. Klin. Ocz..

[31] Merkur A., Damji K.F., Mintsioulis G., Hodge W.G. (2001). Intraocular pressure decrease after phacoemulsification in patients with pseudoexfoliation syndrome. J. Cataract Refract. Surg..

[32] Murdoch I.E., Morris S.S., Cousens S.N. (1998). People and eyes: Statistical approaches in ophthalmology. Br. J. Ophthalmol..

[33] Ying G.S., Maguire M.G., Glynn R., Rosner B. (2018). Tutorial on Biostatistics: Statistical Analysis for Correlated Binary Eye Data. Ophthalmic Epidemiol..

[34] Poley B.J., Lindstrom R.L., Samuelson T.W. (2008). Long-term effects of phacoemulsification with intraocular lens implantation in normotensive and ocular hypertensive eyes. J. Cataract. Refract. Surg..

[35] Mansberger S.L., Gordon M.O., Jampel H., Bhorade A., Brandt J.D., Wilson B., Kass M.A., Ocular Hypertension Treatment Study G. (2012). Reduction in intraocular pressure after cataract extraction: The Ocular Hypertension Treatment Study. Ophthalmology.

[36] Issa S.A., Pacheco J., Mahmood U., Nolan J., Beatty S. (2005). A novel index for predicting intraocular pressure reduction following cataract surgery. Br. J. Ophthalmol..

[37] Abdelghany A.A., Sallam M.A., Ellabban A.A. (2019). Assessment of Ganglion Cell Complex and Peripapillary Retinal Nerve Fiber Layer Changes following Cataract Surgery in Patients with Pseudoexfoliation Glaucoma. J. Ophthalmol..

[38] Burgmuller M., Mihaltz K., Schutze C., Angermann B., Vecsei-Marlovits V. (2018). Assessment of long-term intraocular lens (IOL) decentration and tilt in eyes with pseudoexfoliation syndrome (PES) following cataract surgery. Graefe’s Arch. Clin. Exp. Ophthalmol..

[39] Cimetta D.J., Cimetta A.C. (2008). Intraocular pressure changes after clear corneal phacoemulsification in nonglaucomatous pseudoexfoliation syndrome. Eur. J. Ophthalmol..

[40] Haripriya A., Ramulu P.Y., Chandrashekharan S., Venkatesh R., Narendran K., Shekhar M., Ramakrishnan R., Ravindran R.D., Robin A.L. (2019). The Aravind Pseudoexfoliation Study: Surgical and First-Year Postoperative Results in Eyes without Phacodonesis and Nonmiotic Pupils. Ophthalmology.

[41] Kang E., Park J.H., Yoo C., Kim Y.Y. (2024). Comparison of intraocular pressure fluctuation and glaucoma progression rate between phakic and pseudophakic eyes in pseudoexfoliation glaucoma. Sci. Rep..

[42] Perasalo R. (1997). Phaco-emulsification of cataract in eyes with glaucoma. Acta Ophthalmol. Scand..

[43] Rodriguez-Una I., Fernandez-Vega Cueto A., Rodriguez-Calvo P.P., Garcia M., Fernandez-Vega Cueto L., Cobian-Tovar R., Merayo-Lloves J., Alfonso J.F. (2023). Early Lensectomy in Patients With Pseudoexfoliation: Long-Term Effectiveness and Safety Outcomes. J. Glaucoma.

[44] Pose-Bazarra S., Lopez-Valladares M.J., Lopez-de-Ullibarri I., Azuara-Blanco A. (2023). Feasibility, efficacy and safety of early lens extraction in patients with pseudoexfoliation glaucoma: A feasibility and pilot study. Eye.

[45] Shingleton B.J., Nguyen B.K.C., Eagan E.F., Nagao K., O’Donoghue M.W. (2008). Outcomes of phacoemulsification in fellow eyes of patients with unilateral pseudoexfoliation. Single-surgeon series. J. Cataract Refract. Surg..

[46] Shingleton B.J., Heltzer J., O’Donoghue M.W. (2003). Outcomes of phacoemulsification in patients with and without pseudoexfoliation syndrome. J. Cataract Refract. Surg..

[47] Tekcan H., Mangan M.S., Alpogan O., Imamoglu S., Kose A.O., Ercalik N.Y. (2022). The effect of uneventful cataract surgery in pseudoexfoliation glaucoma with or without previous mitomycin C-augmented trabeculectomy. Int. Ophthalmol..

[48] Zarei R., Azimi A., Fakhraei G., Eslami Y., Naderan M., Nouri-Mahdavi K., Caprioli J. (2022). Combined phacoviscocanalostomy versus phacoemulsification alone in patients with coexisting cataract and mild-to-moderate open-angle glaucoma; a randomized-controlled trial. Eye.

[49] Yalvac I., Airaksinen P.J., Tuulonen A. (1997). Phacoemulsification with and without trabeculectomy in patients with glaucoma. Ophthalmic Surg. Lasers.

[50] Wirbelauer C., Anders N., Pham D.T., Wollensak J., Laqua H. (1998). Intraocular pressure in nonglaucomatous eyes with pseudoexfoliation syndrome after cataract surgery. Ophthalmic Surg. Lasers.

